# Exploring the Drivers of Food Waste in the EU: A Multidimensional Analysis Using Cluster and Neural Network Models

**DOI:** 10.3390/foods14081358

**Published:** 2025-04-15

**Authors:** Anca Antoaneta Vărzaru, Dalia Simion

**Affiliations:** 1 Department of Economics, Accounting and International Business, Faculty of Economics and Business Administration, University of Craiova, 13 A.I. Cuza Street, 200585 Craiova, Romania; 2 Department of Finance, Banking, and Economic Analysis, Faculty of Economics and Business Administration, University of Craiova, 13 A.I. Cuza Street, 200585 Craiova, Romania

**Keywords:** food waste, sustainability, economic development, retail sales, consumer prices, sustainable development goals, European Union

## Abstract

Food waste poses a significant global challenge with profound economic, environmental, and social implications. Within the European Union, socioeconomic conditions, food affordability, and sustainability initiatives create a complex framework for understanding and mitigating food waste. This study examines how economic and sustainability factors shape food waste patterns across EU member states, employing advanced statistical techniques to uncover underlying dynamics. The analysis focuses on five key variables: the Harmonized Index of Consumer Prices for food, food waste, food retail sales, the Sustainable Development Goals Index, and GDP per capita. Factorial analysis and a general linear model were used to investigate linear relationships, and multilayer Perceptron (MLP) neural networks were employed to model the non-linear relationships driving food waste. At the same time, hierarchical cluster analysis identified four distinct country groups, each characterized by unique combinations of these variables. The results reveal that higher GDP per capita and stronger sustainability performance are associated with lower food waste, whereas higher food prices and increased retail activity present more nuanced influences. The findings underscore the importance of customized policies that address the EU’s diverse socioeconomic and sustainability contexts, offering a pathway toward more sustainable food systems and reduced waste.

## 1. Introduction

The global challenge of food waste (FW) has reached critical proportions, casting long shadows across environmental, economic, and social spheres. Within the European Union (EU), food waste presents a paradoxical crisis: despite progressive sustainability policies, the bloc generates approximately 57 million tons of food waste annually, equating to 127 kg per capita [1]. Households represent the largest share of food waste (68%), followed by the manufacture of food products and beverages (23%), restaurants and food services (14%), and retail distribution (10%). Primary production (agriculture, fishing, and aquaculture) contributes 12%, revealing systemic inefficiencies across the entire supply chain, from farm to fork. What makes this issue particularly complex is the complicated net of factors driving wasteful behaviors, where economic realities intersect with social norms and environmental concerns in ways we are only beginning to understand.

This staggering figure underscores the urgency of addressing FW as a core sustainability priority, particularly as the EU aligns with the United Nations’ Sustainable Development Goal Target 12.3 to halve per capita food waste by 2030 [2]. Nevertheless, the path to achieving this goal remains unclear as motivations for change vary dramatically across individuals and communities. For some, the compelling argument comes in cold, complex numbers—the financial losses from wasted food [3]. For others, it is the ethical weight of discarding edible food while millions face hunger or the environmental toll of unnecessary production [4]. This diversity of perspectives suggests that effective solutions must be as nuanced as the people they aim to influence [5].

The EU’s current Farm to Fork Strategy and Circular Economy Action Plan advocate for uniform targets, yet our research reveals significant regional disparities in food waste drivers and patterns. For instance, Eastern European member states face higher post-harvest losses due to infrastructural gaps, while Western Europe’s waste stems predominantly from consumer behavior [1].

While previous studies have examined various pieces of the puzzle, such as economic drivers or environmental impacts, few have captured how these dimensions interact dynamically across different national contexts. This study offers novel insight: a multidimensional map of food waste patterns across the EU using advanced statistical modeling techniques to examine the relationships between food pricing, retail patterns, sustainability metrics, and economic indicators.

The significance of this approach becomes apparent when considering its policy implications. Traditional one-size-fits-all solutions have consistently fallen short because they fail to account for the substantial differences between member states. Some countries struggle primarily with consumer-level waste in affluent societies, while others face systemic losses earlier in the supply chain. Some populations respond strongly to economic incentives, while others require appeals to environmental stewardship. By identifying distinct clusters of countries with similar waste profiles and modeling the non-linear relationships between key variables, this research provides policymakers with a valuable resource: targeted insights that respect regional differences while advancing shared goals.

Methodologically, this study breaks new ground by innovatively combining established analytical techniques. Combining cluster analysis with linear (factorial analysis and general linear model) and non-linear (artificial neural network modeling) represents a promising avenue for revealing broad patterns and subtle national particularities. This dual approach acknowledges a fundamental truth about food systems—they behave as complex adaptive systems where small changes can have disproportionate effects and relationships between variables rarely follow straight lines.

As we stand at the crossroads of multiple global crises—from climate change to food insecurity—understanding and addressing food waste has never been more critical. This research contributes to that understanding by providing a complete picture of the forces shaping European waste patterns.

The article is structured into six main sections. Section 2 presents a literature review and hypothesis development following this introduction. Section 3 describes the materials and methods, followed by the results in Section 4. Section 5 discusses the implications of the findings and future research directions, while Section 6 offers general conclusions.

## 2. Literature Review and Hypothesis Development

### 2.1. Sustainable Consumption and Food Waste

Our choices about food consumption ripple through our societies and ecosystems profoundly and are shaped by an intricate interplay between our inner values and external circumstances. At the most personal level, what we choose to eat—and what we choose to waste—stems from deeply held beliefs about our relationship with the natural world, cultivated through education and lived experience [6,7,8]. Research consistently shows that when individuals develop strong environmental ethics, they are significantly more likely to adopt plant-based diets and minimize food waste, with one study finding a 38% reduction in household waste among environmentally conscious consumers [9]. These values do not emerge in isolation; they are nurtured through thoughtful awareness campaigns that make the consequences of unsustainable consumption tangible, helping people connect daily choices to broader ecological impacts [10]. Particularly compelling is how altruistic concern for others and biospheric concern for the well-being of nature shape sustainable behaviors, from dietary choices to waste reduction practices [11,12].

Nevertheless, our food decisions are never made in a vacuum. Our social worlds exert a powerful influence, especially for younger generations navigating complex digital landscapes where food trends and norms spread rapidly [13]. Economic realities shape our choices equally—when organic produce becomes more affordable through thoughtful subsidies or sustainable options are priced competitively, consumption patterns shift meaningfully [14]. These external factors create a dynamic where personal ethics interact with social and economic structures, sometimes reinforcing and sometimes conflicting with our sustainable aspirations [15].

Governments and businesses contribute decisively to this ecosystem. Thoughtful regulations that bring transparency to food supply chains or incentivize green practices do not just change corporate behavior—they reshape entire markets and consumer expectations [12,16]. Meanwhile, technological innovations are quietly revolutionizing access to sustainable options, with mobile platforms reducing barriers to responsible consumption while building customer loyalty [17,18,19]. The private sector’s evolving approach is particularly noteworthy as companies increasingly recognize that environmental responsibility aligns with long-term viability [20,21]. From transforming food surplus into premium upcycled products to pioneering biodegradable packaging solutions, businesses demonstrate how environmental and economic value can grow together [22].

The ethical dimensions of food waste become painfully apparent when we consider that nearly one-third of all food produced globally is lost or wasted, even as hundreds of millions face hunger [23,24,25,26,27,28,29,30,31]. This represents an economic inefficiency and a moral failure, highlighting deep-seated inequities in resource distribution [32,33]. The complex journey of food from farm to fork to landfill involves countless decision points where waste can occur, each influenced by a unique combination of economic conditions, cultural norms, and policy frameworks [34]. Macroeconomic factors such as food pricing and retail dynamics create distinct waste patterns across different contexts, with affluent societies often wasting more at the consumption stage while developing economies face losses earlier in the supply chain [34,35].

Understanding these interconnected drivers—from individual psychology to global economic forces—is essential for developing solutions that match the complexity of the challenge. The most effective interventions will recognize how personal values, social norms, economic incentives, and policy frameworks interact to shape our relationship with food in all its dimensions.

### 2.2. Drivers of Food Waste

Understanding the drivers behind food waste (FW) has become a critical focal point in sustainability research due to its environmental and economic implications and the methodological complexity associated with capturing its multifaceted nature [29,36,37]. FW does not occur in a vacuum. Socioeconomic dynamics, consumer behavior, institutional frameworks, and technological capacities shape it. Consequently, developing predictive and explanatory models that reflect the realities of food systems necessitates carefully identifying these factors. There is growing consensus in the literature that FW drivers span across individual, institutional, and structural dimensions, and any attempt to address this issue must take into account the interdependence among these layers [29].

Scholars and policymakers advocate for multidimensional approaches that combine economic instruments, technological solutions, and behavioral change initiatives to tackle FW effectively. This perspective is rooted in systems thinking, recognizing that food waste is a systemic issue that requires collaborative engagement across sectors and disciplines. Integrating economic and technological responses with public education and policy design highlights the need for sustained collaboration among researchers, practitioners, and policymakers to develop innovative, socially and environmentally sustainable interventions [29].

From a macroeconomic perspective, FW is associated with several structural indicators influencing consumer behavior and market dynamics. Among these are fluctuations in food prices, retail sales volumes, a country’s economic performance, and the population’s level of awareness regarding waste’s ethical and environmental implications [34,35]. These factors operate synergistically to shape consumption patterns and waste generation, and they are frequently highlighted in theoretical frameworks and empirical studies seeking to model FW generation.

Food price is one of the most widely studied determinants of food waste. Substantial evidence suggests that lower food prices correlate with increased household waste, as affordability often leads to over-purchasing and underutilization of perishable goods [5]. In high-income countries, where food tends to be both accessible and inexpensive, this pattern is particularly pronounced. Studies reveal that overconsumption in these contexts is often accompanied by a diminished awareness of the broader environmental costs associated with discarding edible food [38]. Furthermore, ethical attitudes toward food waste—shaped by values, education, and cultural norms—play an important role in influencing consumer behavior. Individuals with a higher environmental awareness are generally more inclined to manage food purchases and consumption responsibly, thereby reducing waste [39].

The economic burden of FW extends beyond the household. At the national level, waste contributes to inefficiencies in resource allocation and environmental degradation, including increased greenhouse gas emissions and the overuse of water, soil, and energy resources [5,40,41]. These outcomes underline the urgency of developing integrative policy responses that address FW from both macro- and microeconomic perspectives. In this regard, scholars have consistently emphasized the need for strategies that promote behavioral shifts through educational campaigns, financial incentives, and improved access to sustainable consumption alternatives [42].

An additional layer of complexity arises from structural shifts in consumer spending behavior. As societies become wealthy, the proportion of income allocated to food declines, reflecting broader changes in consumption preferences and priorities [43]. Food becomes relatively less valuable, not because of its nutritional worth but due to its diminishing share in the household budget. This perceived decline in value contributes to more casual attitudes toward waste. Consumers in wealthier countries often fail to engage in meal planning or stock management, resulting in the unnecessary disposal of edible food. Targeted awareness campaigns and educational interventions have been recommended to counter this trend, recalibrate food’s perceived value, and encourage sustainable consumption habits [44].

The environmental consequences of modern dietary patterns—particularly those driven by rising incomes and urbanization—are another central theme in the FW literature. A growing global population and increasing demand for resource-intensive foods such as meat and dairy place considerable stress on ecosystems. These food products require disproportionately high land, water, and energy inputs and contribute significantly to greenhouse gas emissions [43]. The resulting environmental degradation, amplified by climate change-induced events such as droughts and floods, further exacerbates the fragility of agricultural systems and food security [45].

Given these interconnections, scholars have advocated for transformative changes to food systems, including dietary shifts toward plant-based foods, the development of resource-efficient agricultural practices, and a concerted effort to minimize FW at all levels of the supply chain [43,45,46]. This call to action aligns with the United Nations’ Sustainable Development Goals (SDGs), particularly SDG 12, which emphasizes “Responsible Consumption and Production”. Within this framework, Target 12.3 aims explicitly to halve per capita FW globally by 2030, recognizing the imperative to align consumer behavior with broader sustainability objectives [47,48].

Achieving this target requires a dual strategy—encouraging consumer behavioral change while improving food distribution’s technological and logistical aspects. Interventions such as enhancing storage capacity, refining transportation infrastructure, and promoting cooking techniques that utilize ingredients more efficiently are all strategies endorsed by the literature [47]. Empirical studies also indicate that countries prioritizing investments in green technologies, education, and stringent FW policies consistently report lower levels of waste [35]. These investments reduce environmental impact and foster a culture of responsibility and stewardship among consumers and producers [5].

From an economic systems perspective, the volume of retail food sales and the overall performance of national economies serve as important contextual variables in understanding FW. The Food and Agriculture Organization (FAO) reports that nearly one-third of global food production is wasted annually, which could feed two billion people [49]. Projections suggest this figure could rise to two-thirds by 2030 [50,51]. In high-income nations, household FW is primarily attributed to factors such as excessive purchasing, poor storage practices, and a general lack of meal planning—behaviors encouraged by the abundant availability and low cost of food [48,52]. Conversely, in low- and middle-income countries, FW is often a consequence of infrastructural deficiencies and the lack of appropriate technologies, especially in the earlier stages of the supply chain [52].

Addressing these disparities requires tailored policy responses and the development of adequate infrastructure. In high-GDP countries, governments typically have greater fiscal and administrative capacity to implement integrated FW policies. Strong economic performance enables investment in modernized storage systems, improved logistics, and public education campaigns informing consumers about waste’s social and environmental costs [53,54,55]. Scholars like Druckman et al. [56] argue that economic growth is a prerequisite for supporting capital-intensive projects needed to reduce FW. These include smart supply chain technologies, digital tracking systems, and robust monitoring frameworks that evaluate intervention outcomes over time.

However, the capacity to implement these interventions varies significantly across countries. Nations with weaker economies often face significant barriers, including limited public funding, human resource shortages, and fragmented governance structures, which impede their ability to pursue effective FW reduction strategies [54,56]. This discrepancy calls for international cooperation and knowledge sharing to help bridge the gap in FW management capabilities across different contexts.

While technological and infrastructural advancements remain vital, the literature emphasizes that effective FW reduction must begin with prevention. Preventive strategies focus on addressing the root causes of waste before they manifest in measurable losses. These include public awareness campaigns, consumer education programs, and policies that reward responsible consumption. In parallel, efforts to repurpose surplus food through redistribution or transformation into value-added products represent a complementary approach, offering economic and social benefits [30,32].

Integrating FW reduction into national food security strategies is now widely seen as an ethical and strategic necessity. Reducing waste improves food availability and helps to alleviate disparities in food access, particularly in regions facing chronic food insecurity [57,58]. In this context, waste reduction is not merely a sustainability goal but a moral imperative supporting the creation of a more equitable and resilient global food system.

This study proposes its first hypothesis, grounded in the interrelationship between macroeconomic indicators and food waste dynamics:

**Hypothesis H1.**  *The Harmonized Index of Consumer Prices for Food (HICP) and the Sustainable Development Goals Index (SDGi) are expected to influence FW negatively, reflecting the role of economic valuation and policy commitment. Conversely, higher retail food sales (RSFs) are hypothesized to exert a positive influence, contributing to increased food waste due to overconsumption and the normalization of disposable consumption practices.*

At the macroeconomic level, HICP reflects food affordability, where higher prices may reduce waste by increasing consumer caution. Conversely, RSF captures retail demand, with elevated sales often linked to overconsumption and waste. Meanwhile, SDGi represents national sustainability efforts, where stronger policy frameworks encourage waste reduction.

**Figure 1:**
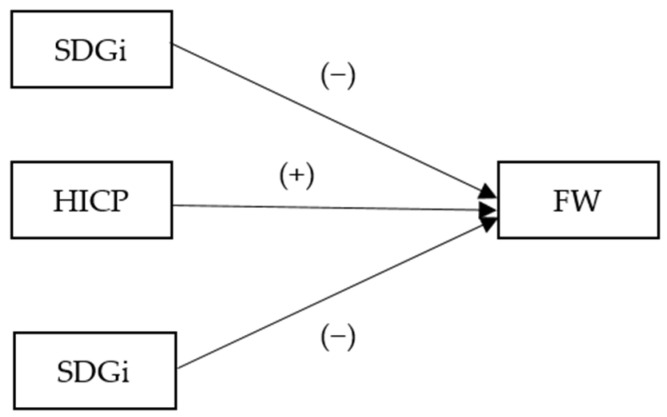
Conceptual model. Source: authors’ design.

The conceptual model (Figure 1) illustrates these interactions.

HICP and SDGi are hypothesized to negatively influence FW as higher prices and sustainability commitments discourage waste. HICP is hypothesized to positively influence FW, as increased retail activity and affluence can lead to excess consumption. The study provides insights into how economic and policy levers can be optimized to reduce food waste across diverse EU contexts by analyzing these relationships.

### 2.3. Differences Between Countries in Food Waste Patterns

Understanding food waste (FW) requires a multidimensional lens integrating socioeconomic, cultural, and infrastructural considerations with established theoretical frameworks. Efforts to reduce FW cannot adopt a one-size-fits-all approach. Instead, they must be tailored to the unique regional and cultural contexts in which they are enacted, acknowledging the variability in waste drivers across different geographic and economic settings [32]. This divergence is rooted in the distinct infrastructural capacities and consumption behaviors that shape how, where, and why food is lost or discarded.

In regions where logistical limitations persist—particularly in developing or rural areas—food losses tend to occur during the production and distribution phases. Fragmentation, inefficiency, and inadequate storage conditions often mark supply chains. Addressing these systemic vulnerabilities requires investment in modern infrastructure, such as refrigerated transport and climate-controlled storage facilities. These technologies preserve food, stabilize local food systems, improve producer incomes, and contribute to regional economic development [32,59]. From a systems theory perspective, interventions at the infrastructure level represent upstream strategies that can yield downstream benefits across the entire value chain.

Conversely, in more urbanized or affluent societies, the dynamics of FW shift substantially. Waste predominantly arises at the consumer level, driven by behavioral patterns rather than technical constraints. The abundance of affordable food, combined with hectic lifestyles and the commodification of food, often leads to over-purchasing, inadequate storage practices, and a lack of meal planning. In such contexts, reducing FW depends less on technological solutions and more on behavioral transformation [26,60]. Public campaigns to raise awareness about FW’s environmental, social, and economic ramifications have demonstrated the potential to reshape consumer habits. Educational initiatives that promote mindfulness, portion control, and the creative use of leftovers can foster more responsible consumption [32]. This approach aligns with behavioral economics frameworks, highlighting the efficacy of “nudging” strategies in influencing routine decision-making.

The role of institutional collaboration cannot be overstated. Coordinated actions among governments, civil society, and private sector actors enhance the scalability and legitimacy of FW interventions. These partnerships are essential to fostering a culture of sustainability and embedding responsible consumption practices into everyday life.

Across the European Union, the issue of FW has emerged as a pressing policy concern. Despite shared membership in a supranational framework, EU countries exhibit notable disparities in the causes and intensity of FW. In high-income member states, household waste levels remain alarmingly high. This situation is paradoxical, as higher GDP per capita typically correlates with greater access to education and awareness regarding waste’s environmental and ethical implications [61,62]. Theoretically, affluence should promote conscientious consumption; however, empirical evidence suggests the opposite. Increased wealth, rather than promoting restraint, often leads to overconsumption. The relative affordability of food diminishes its perceived value, reinforcing habits of discarding rather than conserving [62].

This behavioral tendency underscores a disjunction between economic development and sustainability orientation. While countries with robust economies possess the fiscal and institutional capacity to invest in sustainable technologies and public education, these advantages do not automatically translate into lower FW levels unless accompanied by deliberate policy measures and cultural shifts [63]. Measures that encourage meal planning, proper food storage, and ingredient optimization can mitigate these patterns. More importantly, institutional support through policies that incentivize sustainable practices and penalize excessive waste could serve as a corrective mechanism.

A deeper understanding of these dynamics enables researchers to detect structural patterns and formulate more nuanced hypotheses. Drawing from sustainability transition theory and institutional theory, it becomes apparent that socio-cultural and economic dimensions play a pivotal role in shaping FW behaviors across different regions. The intersection of key indicators—food prices, FW levels, retail sales, sustainability orientation, and economic performance—can thus be leveraged to identify country clusters with shared characteristics.

In light of these insights, this study proposes a second hypothesis that reflects a classification logic based on empirical regularities:

**Hypothesis H2.**  *EU countries can be grouped into homogeneous clusters based on HICP, FW, RSF, SDGi, and GDPpc, reflecting shared patterns in food prices, waste, retail sales, sustainability, and economic development, with higher GDPpc and SDGi linked to lower FW and more efficient RSF.*

This hypothesis builds on the notion that macro-level indicators are not merely descriptive but can also serve as proxies for more profound structural configurations that shape food systems. In turn, identifying these configurations allows policymakers to design targeted interventions that align with each country’s specific stage of development and socioeconomic context. The clustering approach acknowledges the heterogeneity within the EU and enables more customized, practical strategies for addressing FW, reinforcing the broader objectives of the European Green Deal and the UN Sustainable Development Goals.

## 3. Materials and Methods

### 3.1. Research Design

Based on essential food prices, waste, retail sales, sustainability, and economic development measures, the study was intended to investigate the complex interactions and groupings across EU countries. A quantitative approach was adopted to achieve this, leveraging advanced statistical methods to uncover patterns and structures within the data. The research design was structured around two primary analytical techniques: cluster analysis and artificial neural network analysis, specifically Multilayer Perceptron (MLP). Cluster analysis was employed to identify homogeneous groups of countries sharing similar characteristics, while MLP was used to model complex, non-linear relationships between the variables. This dual-method approach ensured a comprehensive exploration of the hypothesis, combining both exploratory and predictive methods to uncover meaningful insights and validate the findings.

The research design was guided by the need to address the complexity of food systems and their links with economic and sustainability factors. By focusing on EU countries, the study aimed to capture the diversity of socioeconomic conditions and regional policy frameworks. Using cluster analysis allows for identifying natural groupings within the data, revealing patterns that might not be immediately apparent through traditional analytical methods. Meanwhile, the MLP approach provided a robust framework for modeling the intricate dependencies between variables, offering predictive insights into how changes in one factor might influence others. These methods ensured an integrated understanding of the factors driving FW, retail behavior, and sustainability efforts across the EU.

### 3.2. Selected Variables

The analysis utilized five key variables, each capturing a distinct aspect of the socioeconomic and sustainability landscape within the EU. HICP reflects the annual average price changes for food products, measuring affordability and inflationary pressures. This index is particularly relevant for understanding how price fluctuations influence consumer behavior and food accessibility, as higher food prices may lead to more cautious consumption patterns or exacerbate food insecurity in vulnerable populations. By incorporating HICP, the study captures the economic dimension of food systems, providing a lens through which to examine the relationships between affordability and FW.

FW quantifies the bio-waste generated by households and similar sources. High levels of FW may signal inefficiencies in food production, distribution, or consumption, while lower levels could reflect effective waste management practices or more responsible consumer behavior. By analyzing FW, the study sheds light on food consumption’s environmental and social implications across the EU.

Retail sales of food (RSF), indexed to 2021 levels, capture trends in food purchasing behavior and market dynamics. This variable provides a snapshot of consumer demand and retail activity, reflecting how economic conditions, cultural preferences, and market structures influence food consumption. Higher RSF values may indicate robust consumer spending or a preference for convenience foods, whereas lower values could suggest economic constraints or a shift toward alternative food sources. By examining RSF, the study explores the economic drivers of food systems and their potential impact on sustainability outcomes.

The Sustainable Development Goals Index (SDGi) offers a composite score reflecting a country’s progress toward achieving global sustainability targets, encompassing environmental, social, and economic dimensions. This index is a comprehensive measure of a nation’s commitment to sustainability, capturing efforts to address climate change, reduce inequality, and promote responsible consumption. A high SDGi score indicates strong performance across multiple sustainability indicators, while a lower score may highlight areas needing improvement. By integrating SDGi into the analysis, the study evaluates how broader sustainability efforts intersect with food systems and waste management practices.

GDP per capita in Purchasing Power Standards (GDPpc) measures economic prosperity, adjusted for price level differences across countries, and is a proxy for overall living standards. This variable provides a critical context for understanding the economic capacity of EU countries, as higher GDPpc levels often correlate with greater resources for sustainability initiatives and waste reduction programs. Conversely, lower GDPpc levels may indicate economic challenges that constrain efforts to address FW or improve food system efficiency. By including GDPpc, the study highlights the role of economic development in shaping food systems and sustainability outcomes.

These variables provide an integrated view of the factors influencing food systems and sustainability across the EU. They capture food consumption and waste’s economic, environmental, and social dimensions, offering a comprehensive framework. This multidimensional approach ensures that the study addresses the diverse challenges and opportunities within EU food systems, contributing to a deeper understanding of how these factors interact and how they can be leveraged to promote more sustainable and resilient food systems.

Table 1 presents the variables used in empirical research.

Table 2 presents the descriptive statistics for the variables analyzed in this study, including the minimum, maximum, mean, standard deviation, skewness, and kurtosis values. These statistics provide a foundational understanding of the data distribution and variability across the selected indicators.

HICP ranges from 92.5 to 163.54, with a mean of 137.173 and a relatively low standard deviation of 12.4735, indicating moderate variability across EU countries. FW shows a wide range, from 65 to 294 kg per capita, with a mean of 137.173 and a high standard deviation of 47.2149, reflecting significant disparities in waste generation. The positive skewness (1.232) and high kurtosis (2.281) for FW indicate a right-skewed distribution characterized by a concentration of lower values and a few outliers with exceptionally high waste levels.

Retail sales of food (RSF) exhibit a relatively narrow range, from 91.3 to 124.2, indicating consistent retail behavior across the EU. The Sustainable Development Goals Index (SDGi) ranges from 72.5 to 86.8, with a mean of 80.125, reflecting moderate variability in sustainability performance. GDP per capita (GDPpc) ranges from 57 to 260, with a mean of 104 and a high standard deviation of 44.138, highlighting significant regional economic disparities. The positive skewness (2.214) and high kurtosis (5.047) for GDPpc indicate a right-skewed distribution with a concentration of lower-income countries and a few high-income outliers.

The descriptive statistics reveal notable variability across the variables, particularly in food waste and GDP per capita, underscoring EU countries’ diverse socioeconomic and sustainability profiles. These findings provide a context for interpreting factorial analysis, the general linear model, MLP analysis, and cluster results, highlighting the need for customized policies to address the unique challenges and opportunities within different segments of the EU.

### 3.3. Methods

Four statistical methods were employed to analyze the data: factorial analysis, a general linear model for linear relationships, artificial neural network analysis (MLP) for non-linear relationships, and hierarchical cluster analysis to group countries depending on their characteristics.

Factor analysis allowed us to uncover latent structures—those underlying dimensions that subtly shape the interaction among the observed variables. This method acted much like an X-ray of our correlation matrix, helping to reveal the extent to which the shared variance across variables could be attributed to deeper, hidden factors. We employed principal component extraction to explain the maximum total variability using the fewest possible dimensions to achieve this. The foundational equation for this approach can be expressed as follows:

(1) X=AF+∈

X—observed variables (SDGi, HICP, FW, RSF).

A—factor loadings.

F—principal components (extracted common factors).

∈—errors.

A general linear model (GLM) was employed to examine linear relationships between the dependent variable (FW) and a set of independent predictors (HICP, RSF, SDGi) The foundational equation for this approach can be expressed as follows:

(2) $$FW=\beta_{0}+\beta_{1}{HICP}_{1}+\beta_{2}{RSF}_{2}+\beta_{3}{SDGi}_{3}+\in$$

FW—dependent variable.

$HICP,{RSF}_{2},{SDGi}_{3}$—independent variables.

$\beta_{0}$—intercept.

$\beta_{1},\beta_{2},\ldots ,\beta_{n}$—the regression coefficients.

∈—errors.

This modeling strategy enabled us to test multiple predictors simultaneously against a single outcome variable, offering a comprehensive perspective on the relationships at play. We estimated the model parameters using the least-squares method and assessed their statistical significance through t-tests. To evaluate the model’s explanatory power, we examined the R^2^ value. Additionally, we visually inspected the assumptions of linearity, homoscedasticity, and normality of residuals to ensure the robustness and validity of the findings.

The Multilayer Perceptron (MLP) approach was used to model the complex, non-linear relationships between the variables. MLP is an artificial neural network comprising multiple layers of interconnected nodes, or neurons, that process input data to produce output predictions [IBM]. The network architecture typically includes an input layer, one or more hidden layers, and an output layer (3):

(3) $$y=(\sum_{i=1}^{n}w_{i}x_{i}+b)=\varphi (W^{T}X+b)$$

y—vectors of outputs.

w, x—vectors of weights and inputs.

b—bias.

i—cases.

φ—activation functions.

The activation function used in the hidden layers was the sigmoid function, defined as (4):

(4) $$f\left(n\right)=\frac{1}{1+e^{-n}}$$

n—input variables (EU countries values for SDGi, HICP, and RSF).

f(n)—output variables (EU countries values for FW).

This function allows the network to capture non-linear relationships between variables, particularly suited for modeling complex systems like food waste and sustainability [68]. The MLP model was trained using a scaled conjugate gradient optimization algorithm, which adjusts the network weights to minimize the error between predicted and actual values.

Hierarchical cluster analysis was used to group countries based on their similarity across the selected variables [69]. This method begins by treating each country as a separate cluster and iteratively merges the most similar pairs until a predefined stopping criterion is met. The distance between clusters was calculated using the Squared Euclidean distance [70]. The optimal approach was the average linkage method between groups (5):

(5) $$d_{ij}=\frac{1}{kl}\sum_{i=1}^{k}\sum_{j=1}^{l}d(X_{i},Y_{j})$$

$X_{1,}X_{2,},\ldots ,X_{k}$—observations from cluster 1.

$Y_{1,}Y_{2,},\ldots ,Y_{l}$—observations from cluster 2.

d(X,Y)—distance between a subject with observation vector x and a subject with observation vector.

k, l—cases (EU countries values for SDGi, HICP, FW, and RSF).

The resulting dendrogram visually represents the clustering process, illustrating the hierarchical relationships between countries.

Together, these methods provided a robust framework for exploring the data, validating the hypothesis, and drawing meaningful conclusions about the relationships between food waste, sustainability, and economic development in the EU. Combining cluster analysis with factorial analysis, the general linear model, and MLP uncovered broad patterns and specific insights, offering a comprehensive understanding of the factors driving food waste and sustainability across the region.

## 4. Results

The factor analysis conducted on the four key variables—the Harmonized Index of Consumer Prices (HICP), food waste (FW), retail sales of food (RSF), and the Sustainable Development Goal Index (SDGi)—revealed several important insights into how these indicators interact and how they can cluster into shared dimensions. The results indicated an underlying structure that captures economic dynamics and sustainability concerns, although some relationships appeared more prominent than others.

The correlation matrix highlighted significant relationships between particular variables, while others lacked strong associations. A notably high positive correlation emerged between HICP and RSF (0.750), indicating that food prices and retail sales tend to move in the same direction, likely driven by market mechanisms. In contrast, FW showed a moderate negative correlation with both HICP (−0.251) and SDGi (−0.307), suggesting that inflationary pressures and stronger sustainability performance may contribute to lower levels of food waste (Table 3).

The Kaiser–Meyer–Olkin (KMO) test returned a value of 0.392, which, although falling below the commonly accepted threshold of 0.6, still provided some justification for conducting factor analysis, albeit with certain limitations. At the same time, Bartlett’s test yielded a statistically significant result (*p* < 0.001), confirming that the correlation matrix differs sufficiently from an identity matrix and thus supports further analysis.

The communalities, which measure the proportion of each variable’s variance that the common factors can explain, ranged from 0.673 for FW to 0.897 for HICP. These values showed that a substantial portion of the variability in HICP and RSF aligned with the extracted factors, whereas FW and SDGi exhibited lower factor loadings, possibly indicating the influence of additional variables not included in the current model (Table 4).

Principal component analysis extracted two significant dimensions that explained 77.33% of the total variance (Table 5).

The first component accounted for 45.19% of the variance and was strongly defined by HICP (0.937) and RSF (0.893). This grouping suggests a shared economic dimension centered on pricing trends and retail activity, likely capturing the dynamics of the food market where fluctuations in price directly influence consumer purchasing behaviors.

The second component explained 32.14% of the total variance and was primarily associated with FW (0.736) and SDGi (−0.830). This contrast reflected a tension between food waste and sustainability performance: countries with higher SDGi scores generally reported lower levels of food waste, while those with elevated FW levels tended to score lower regarding sustainability. Interestingly, the SDGi exhibited a strong negative loading, indicating its role as a potential mitigating factor against food waste.

The findings suggest that the four variables naturally group into two distinct dimensions: economic (HICP and RSF) and sustainability-related (FW and SDGi). This distinction may have important implications for public policy, highlighting the value of integrated strategies that address market conditions and sustainable development goals. While the factor analysis revealed meaningful patterns within the data, it also underscored the need for further investigation to fully grasp the complex interactions among food prices, waste levels, retail behavior, and sustainability commitments. Even so, the results provide a strong analytical foundation for future discussions on optimizing policy interventions that balance economic efficiency and environmental responsibility.

The univariate GLM analysis offered a structured approach to examine one dependent variable—food waste (FW)—concerning several independent variables and their interactions. Table 6 presents a detailed overview of how three explanatory variables—the HICP (Harmonized Index of Consumer Prices), RSF (Retail Sales of Food), and SDGi (Sustainable Development Goals Index)—affect levels of food waste.

The statistical model is significant, as indicated by an F-value of 8.084 and a *p*-value of 0.000. This result confirms that, taken together, the independent variables meaningfully explain variations in food waste. The coefficient of determination, R^2^ = 0.240 (adjusted R^2^ = 0.210), suggests that the three predictors explain roughly 24% of the total variance in food waste. This percentage holds considerable weight, especially given the complexity of consumer behavior and the broader socioeconomic context surrounding food consumption.

Beyond the model as a whole, each variable underwent an individual evaluation to assess its distinct contribution to explaining food waste. The intercept, which represents the estimated level of waste when all predictors equal zero, is statistically significant (F = 16.551, *p* = 0.000). While this value does not offer direct practical relevance—since real-world values for HICP, RSF, and SDGi rarely approach zero—it does serve as a baseline for understanding the estimated coefficients within the model’s framework.

When focusing on HICP, the analysis reveals a statistically significant impact on food waste (F = 14.498, *p* = 0.000). The partial eta squared value of 0.158 indicates that HICP accounts for approximately 15.8% of the variation in food waste, even after controlling for the other predictors. This strong and clear association suggests that higher consumer prices may drive households to reduce waste, likely due to increased financial pressure that promotes more thoughtful purchasing and consumption.

RSF, which reflects the volume of food sold through retail, also significantly influences food waste, albeit to a lesser extent. With an F-value of 6.590 and a *p*-value of 0.012, RSF emerges as a significant predictor. The corresponding partial eta squared of 0.079 indicates that it explains about 7.9% of the variation in food waste, independent of other factors. This result aligns with patterns often observed in economies where increased food availability and aggressive marketing can lead to overconsumption and, subsequently, higher levels of waste.

Among the most compelling findings, the SDGi shows a strong negative effect on food waste (F = 13.582, *p* = 0.000), with a partial eta squared of 0.150. This result indicates that approximately 15% of the observed variation in food waste is correlated with a country’s achievement of the Sustainable Development Goals. Countries that score higher on sustainability indicators tend to generate less food waste. This relationship suggests that the SDGi captures more than just environmental or economic policies—it also reflects public awareness, waste management efficiency, civic initiatives, and supportive infrastructure. A higher SDGi score typically indicates a societal ecosystem that prioritizes sustainable practices and incorporates food waste reduction into its policies and daily routines.

Examining the individual parameter coefficients reveals that all three independent variables contribute statistically significantly to the dependent variable (Table 7).

HICP shows a significant negative coefficient (B = −2.196, *p* < 0.001), indicating that per capita food waste decreases as food prices rise. Consumers likely adjust their behavior when prices increase, becoming more cautious about what and how much they purchase and using food more efficiently. This pattern reflects a kind of economic self-regulation that discourages waste through financial constraints—a phenomenon well-documented in the literature, particularly regarding the inverse relationship between price increases and impulsive or excessive buying.

In contrast, RSF has a positive and significant coefficient (B = 2.716, *p* = 0.012). This result suggests that higher volumes of retail food sales are associated with greater levels of waste. A more abundant and accessible food supply may lead consumers to buy more than necessary, increasing the likelihood of spoilage before consumption. This phenomenon is evident in highly developed economies, where overconsumption and marketing practices drive a substantial portion of household food waste.

Perhaps the most revealing result lies in the SDGi’s negative coefficient (B = −5.477, *p* < 0.001). This finding highlights a clear relationship: countries that excel in meeting sustainability goals also tend to waste less food. SDGi captures various factors, including environmental, economic, and social dimensions. It reflects national policies, public engagement, waste infrastructure, and civic awareness. Therefore, a higher SDGi score indicates a societal model that prioritizes sustainability, where food waste is actively addressed both institutionally and culturally.

The statistical significance of the three predictors is further supported by the partial eta squared values: HICP (0.158), SDGi (0.150), and RSF (0.079). These values quantify the proportion of variation in the dependent variable (food waste) explained by each predictor, accounting for the presence of the others in the model. The observed power values reinforce confidence in the findings, as all exceed the conventional threshold of 0.70. HICP (0.964) and SDGi (0.953) reach exceptionally high levels. This result indicates that the model will likely detect actual effects within the sample, and the results are based on solid statistical ground.

Overall, the general linear model illustrates a nuanced interplay between economic, commercial, and sustainability-related factors in shaping food waste outcomes. Rising food prices and stronger sustainability performance contribute to reducing waste, while increased retail activity—absent effective consumer education or policy safeguards—may amplify it. These insights lay the groundwork for integrated public policy, highlighting the importance of promoting responsible consumption, supporting initiatives to reduce food waste, and strengthening the connection between sustainability goals and everyday consumer behavior.

**Figure 2:**
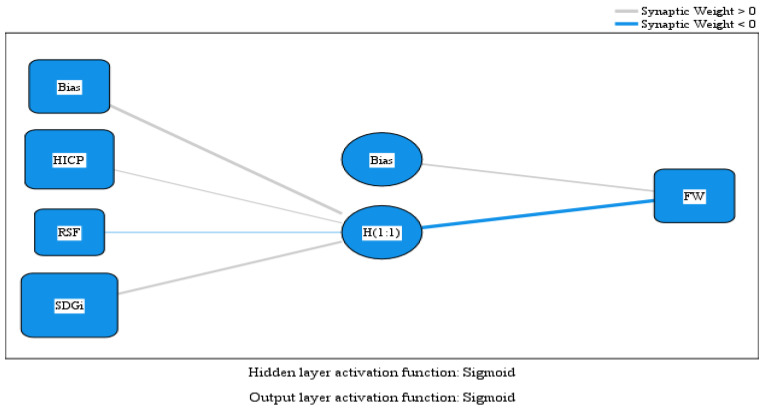
MLP model. Source: authors’ design using SPSS v.27 (IBM Corporation, Armonk, NY, USA).

In the context of the Hypothesis H1 investigation, the Multilayer Perceptron (MLP) neural network model was employed to explore the non-linear relationships between the variables HICP (Harmonized Index of Consumer Prices for food), RSF (Retail Sales of Food), SDGi (Sustainable Development Goals Index), and FW (Food Waste). Figure 2 illustrates the relationships within the MLP model, while Table 8 presents the estimated parameters of the model.

The Multilayer Perceptron (MLP) model analysis offers detailed insights into the relationships between variables and their contributions to explaining FW, enabling a deeper interpretation of the results. The positive influence of HICP and SDGi on reducing FW is particularly noteworthy. HICP has a positive coefficient of 1.054 in the hidden layer, indicating a significant impact on reducing FW. This result supports the hypothesis that increasing food prices could lead to a more responsible use of food resources. For instance, when food prices rise, consumers may become more aware of the value of food, thereby reducing the amount wasted. This mechanism aligns with observations in the literature, which suggest that higher prices can encourage more economical and sustainable behavior.

Regarding SDGi, it has the highest positive coefficient (1.306) among all variables, confirming its essential role in reducing FW. The Sustainable Development Goals Index measures progress in sustainability, resource management, and education, all directly linked to waste reduction. For example, countries with high SDGi scores tend to implement policies and programs that promote resource efficiency and FW reduction. Thus, the positive influence of SDGi aligns with hypothesis H1, confirming that progress in achieving sustainable development goals contributes to diminishing FW.

On the other hand, RSF (retail sales of food) has a negative coefficient of −0.266 in the hidden layer, suggesting a negative influence on FW. This result indicates that increased retail sales could lead to higher FW. This finding might be explained by the fact that greater availability of food products at the retail level could encourage overconsumption or inefficient stock management, leading to increased waste. For instance, retail chains offering promotions or discounts on large quantities of products might incentivize excessive purchasing, resulting in food that goes uneaten and is ultimately wasted.

Hypothesis H1 is supported by the model’s data, which show that HICP and SDGi have significant positive coefficients (1.054 and 1.306, respectively), confirming their positive influence on reducing FW (given the negative influence of the hidden layer on FH). Meanwhile, RSF has a negative coefficient (−0.266), confirming its negative influence on increasing FW. The variable importance analysis reveals that SDGi has the highest normalized importance (100%), followed by HICP (76.7%) and RSF (17.0%). This underscores that SDGi is the strongest predictor of FW, followed by HICP, while RSF has a smaller yet significant influence.

The linear and non-linear models validate hypothesis H1. The findings highlight the importance of addressing economic and sustainability factors to tackle the global challenge of FW effectively.

**Figure 3:**
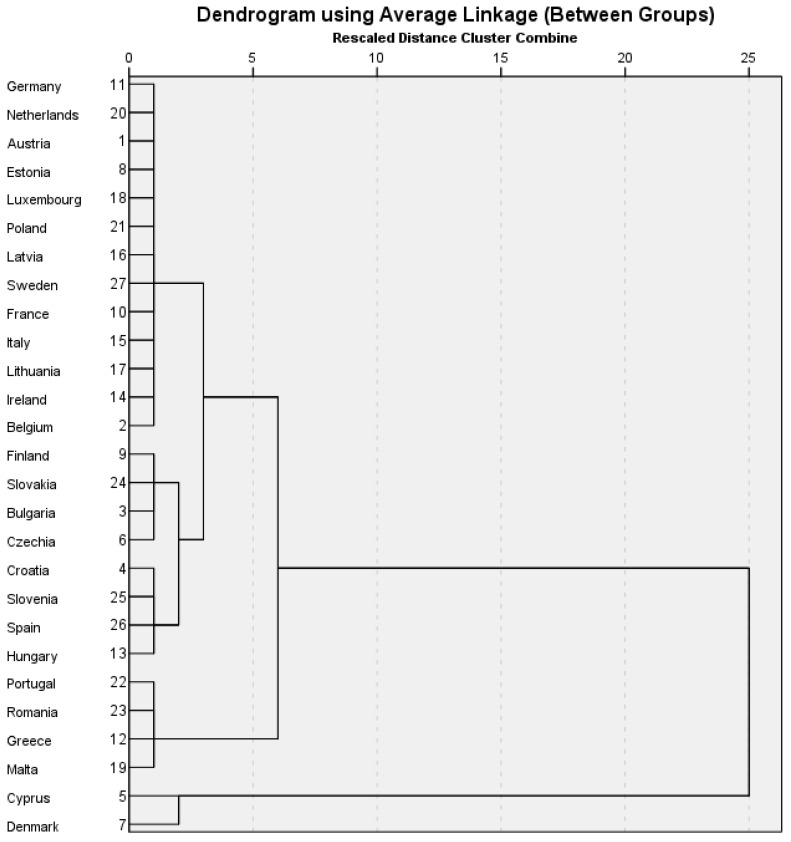
Dendrogram. Source: authors’ design using SPSS v.27 (IBM Corporation, Armonk, NY, USA).

We employed cluster analysis to investigate Hypothesis H2, which posits that EU countries can be grouped into homogeneous clusters based on their performance across key indicators such as HICP, FW, RSF, SDGi, and GDPpc. This method identifies natural groupings within the data, revealing patterns and relationships that might remain hidden across the European Union. Figure 3 presents the resulting dendrogram from the cluster analysis, illustrating the hierarchical relationships between countries and the formation of distinct clusters based on their shared characteristics.

Following the cluster analysis, a more refined understanding emerges regarding how European Union member states align according to key performance indicators such as the Harmonized Index of Consumer Prices (HICP), food waste (FW), retail sales figures (RSF), Sustainable Development Goals index (SDGi), and GDP per capita (GDPpc). The data uncover four distinct clusters (see Table A1 in the Appendix A), each characterized by specific economic, social, and sustainability profiles, which provide essential insights into national-level patterns of food system efficiency and sustainability.

The first cluster, comprising countries such as Austria, Germany, and Sweden, is characterized by a relatively high GDP per capita and a robust performance in achieving sustainable development goals. These nations also maintain moderate levels of food waste and demonstrate efficiency in retail food distribution. For instance, Belgium, with a total food waste of 151 kg per capita, surpasses the EU mean and its cluster’s average. A closer look into sectoral contributions indicates that an overwhelming portion—63 kg—is generated during manufacturing. This value is substantially higher than the EU average for that sector, pointing to potential inefficiencies in the industrial food processing chain. On the other hand, despite its strong economic standing and well-developed food systems, Germany reports a more moderate total of 129 kg per capita. The distribution of waste here is more balanced, with household food waste (75 kg) forming the majority, which suggests that public awareness campaigns and consumer-level interventions might be particularly impactful in this context [1].

Another interesting case within this cluster is Ireland, where the total food waste per capita is 144 kg. Unlike Belgium, Ireland’s food waste appears to be more evenly spread across sectors, though households contribute a relatively more minor share (42 kg) than the EU average. What stands out is the considerable amount of waste generated by the food service sector (30 kg), highlighting potential overproduction and inefficiencies in the hospitality industry [1].

In contrast, the second cluster, composed of countries such as Bulgaria, Hungary, and Spain, presents a markedly different picture. In Bulgaria, the total food waste is just 95 kg per capita, significantly below the EU average. The household sector contributes 41 kg to this figure, which is relatively modest but still forms the largest share. Manufacturing waste is also relatively low at 23 kg, which may reflect a combination of smaller-scale food production and perhaps underreporting or limited data availability. A similar pattern is visible in Hungary, where total food waste is even lower, at 84 kg per capita. Here, household waste makes up the lion’s share (60 kg), with negligible contributions from other sectors such as food services (2 kg) [1]. These numbers highlight a general trend of lower overall consumption and industrial activity, which aligns with these countries’ more modest GDP per capita and lower consumer price levels.

The nations from the second cluster report lower GDP per capita and SDGi scores than the first group but also display significantly lower levels of food waste. This inverse relationship suggests that economic constraints may encourage more cautious consumption habits, potentially reducing food waste. However, the elevated HICP values observed in this cluster signal a persistent issue with food affordability. Thus, while reduced waste might be seen as a positive outcome, it occurs within a broader context of limited purchasing power and constrained access, raising concerns about food security and equity.

Cluster three, which includes Cyprus and Denmark, presents a notable paradox. Both countries have high GDP per capita and have made substantial progress toward their sustainability goals; however, they also report the highest food waste levels within the EU sample, particularly pronounced in Cyprus. Cyprus emerges as an outlier with an alarming 294 kg of food waste per capita—the highest in the EU by a wide margin. A detailed breakdown reveals that waste is generated across all stages of the food supply chain, with the highest levels reported in manufacturing (72 kg), retail and distribution (60 kg), and households (77 kg). Notably, primary production contributes significantly (52 kg), indicating systemic inefficiencies from the beginning of the food value chain. These figures raise concerns about resource management, regulatory enforcement, and perhaps structural issues within Cyprus’s agri-food systems. In Denmark, food waste is similarly elevated at 254 kg per capita, but the nature of the problem differs. Manufacturing accounts for nearly half of this figure (118 kg), reflecting an industrial-scale food system that, while efficient in output, seems to struggle with surplus management and loss mitigation [1].

Cultural attitudes, consumer behavior, and structural inefficiencies within the food supply chain may all undermine waste reduction efforts in these countries, even in advanced and policy-driven environments. This cluster highlights the importance of examining quantitative and qualitative indicators influencing food system outcomes.

The fourth cluster, comprising Greece, Malta, and Romania, exhibits moderate economic performance and relatively average SDGi achievements but is marked by persistently high levels of food waste. Romania, for example, reports 181 kg of food waste per capita. Households are the main contributors (99 kg), but primary production (32 kg) and food services (29 kg) also play significant roles. These figures may reflect gaps in food preservation infrastructure, inefficient supply chain management, and a lack of coordinated national policies targeting food waste. Similarly, Portugal records 184 kg per capita, with an exceptionally high proportion (123 kg) generated by households [1]. These values suggest a culturally embedded pattern of over-purchasing or insufficient food planning at the consumer level, even without high purchasing power.

This cluster pattern indicates systemic challenges in aligning sustainability goals with concrete food production, distribution, and consumption practices. Despite ongoing policy efforts, these countries may face institutional, infrastructural, or behavioral barriers that hinder progress toward reducing food waste. Consequently, this cluster reflects the need for more tailored and context-sensitive strategies that address the structural and cultural factors contributing to inefficiencies.

The cluster analysis results support Hypothesis H2, confirming that EU countries can be classified into coherent groupings based on their economic indicators, sustainability achievements, food pricing dynamics, and waste generation patterns. Notably, the variation across clusters underscores the heterogeneous nature of the European food landscape, where different countries face distinct challenges and opportunities. These insights affirm the necessity of avoiding one-size-fits-all approaches. Instead, there is a pressing need for nuanced, cluster-specific policy designs that account for local socioeconomic conditions, institutional capacities, and cultural practices.

From a policy-making perspective, this analytical framework offers a valuable tool for designing and implementing more effective strategies tailored to the realities of each cluster. In higher-income countries with advanced sustainability records but persistent food waste, efforts might focus on shifting cultural attitudes and strengthening supply chain accountability. Meanwhile, in lower-income regions where food affordability remains a concern despite lower waste levels, interventions could prioritize access, affordability, and infrastructural improvements to ensure resilience without exacerbating waste. For mid-performing countries struggling with waste and sustainability performance, holistic approaches integrating education, investment, and innovation will be essential.

By identifying these differentiated pathways, the study enhances the empirical understanding of food system dynamics across the EU and provides actionable insights for shaping the next generation of sustainability policies. These findings can inform EU-wide strategies to reduce food waste and promote equitable, sustainable development across all member states.

## 5. Discussion

FW is not merely an issue of economic efficiency but one with profound implications for ecosystems [25,27,71]. This aspect underscores the necessity for an integrated approach that considers both the human and ecological dimensions of the problem.

In this study, we explored the complex relationships between food prices, FW, retail sales, sustainability, and economic development in EU countries using two primary methods: cluster analysis and artificial neural networks (MLP). The results validated the two main hypotheses of the study, offering a comprehensive perspective on factors influencing FW and the sustainability of food systems in the EU.

Hypothesis H1 was validated through the MLP model, demonstrating that the Harmonized HICP and SDGi significantly impact reducing FW. These results align with previous research, highlighting that higher food prices can stimulate more responsible consumer behavior, thereby reducing waste [42,43]. SDGi, as a measure of progress in sustainability, was identified as the strongest predictor of FW reduction, confirming the importance of sustainability policies and inefficient food resource management [44].

However, our results contrast Gencia and Balan’s [55] results, who demonstrated that countries with developed economies do not always generate less FW [72,73,74,75]. They emphasized that easy access to resources and high living standards can lead to less responsible consumption, amplifying FW. This suggests that while macroeconomic indicators and sustainability policies matter, cultural and behavioral factors are essential in determining FW levels. Therefore, a multidimensional approach integrating economic and socio-cultural factors is necessary [76,77,78].

Hypothesis H2 was validated through cluster analysis, which identified four distinct groups of EU countries based on their HICP, FW, retail sales, SDGi, and GDP per capita performance. These clusters highlighted the socioeconomic and sustainability diversity within the EU, emphasizing the need for policies customized to each group’s specifics. For example, clusters with stronger economies and better sustainability performance (such as Austria, Germany, and Sweden) demonstrated more responsible practices regarding consumption and FW management. On the other hand, clusters with weaker economies (such as Bulgaria and Hungary) showed lower levels of FW, likely due to more prudent consumption patterns, but highlighted challenges related to food accessibility.

These results align with studies by Stöckli et al. [79] and Reynolds et al. [80], highlighting the importance of approaches adapted to local contexts to achieve sustainable results in reducing FW [5,81,82]. Strategies for lowering FW promote environmental sustainability and contribute to ensuring food security. By optimizing supply chains and educating consumers about efficient food resource management, significant benefits can be achieved for the environment and society [25]. Approaches such as the circular economy, which promotes resource reuse and recycling, minimize FW’s environmental impact [71].

Managing FW must involve interconnected strategies: identifying the causes of FW [83], stakeholder awareness of FW and combating this phenomenon [84], optimizing food production processes [85], improving packaging and labeling, more efficient distribution of food resources [86], analyzing production and consumption systems through econometric tools [87], and integrating circular economy principles into the agrifood chain, which can lead to a more sustainable system where resources are recycled and reused optimally [26,59].

Recent studies have shown that efficient household FW management represents a key point in reducing FW. Consumers who plan meals, creatively store food, and adequately use leftovers can significantly minimize this phenomenon [88,89]. Therefore, education and public awareness are essential to promoting sustainable behavioral changes and reducing the impact of FW on society and the environment.

### 5.1. Theoretical Implications

FW cannot be attributed exclusively to specific social or demographic categories or strictly correlated with a country’s GDP. It represents a universal human behavior manifested in European regions and globally, regardless of the population’s economic status or demographic structure.

One of the main theoretical contributions of this study is the integration of economic variables (such as HICP and GDP per capita) with sustainability indicators (SDGi) to explain FW. This approach emphasizes that FW cannot be understood solely through isolated economic or environmental factors but requires an integrated analysis that accounts for complex interactions between these dimensions. The results confirm that SDG progress reduces FW, supporting the need for an integrated approach in sustainability policies.

The study confirms the hypothesis that higher prices can lead to reduced FW as consumers become more aware of the value of food resources. This finding aligns with economic theories suggesting that higher prices can stimulate more responsible behavior. However, our study introduces important nuance, highlighting that this effect is not universal and may be influenced by cultural and socioeconomic factors.

The cluster analysis results highlighted that countries with strong economies and high sustainability performance do not always generate the smallest FW. This finding contributes to the literature by highlighting the importance of integrating cultural factors into FW analysis models.

Although there are variations in the extent and causes of FW, this problem is not specific only to certain social groups or economies. In developed countries, waste is often generated at the consumer level through excessive product acquisition or a lack of efficient food preservation practices. In contrast, in emerging economies, losses frequently occur in the initial stages of the supply chain due to deficient storage and distribution infrastructure. Thus, combating FW requires a global approach based on a deep understanding of the diversity of factors involved and implementing strategies adapted to each socioeconomic and cultural context.

### 5.2. Practical Implications

Addressing FW is not merely an economic and social necessity but also a moral and ecological imperative. Implementing effective strategies to prevent and reduce FW could lead to a more sustainable agrifood system that respects human needs and planetary boundaries. The findings of this study carry significant implications for public policies and private initiatives aimed at curbing FW and promoting sustainability.

First and foremost, FW is not just a matter of economic efficiency but has profound implications for ecosystems. Therefore, an integrated approach is necessary considering the issue’s human and ecological dimensions. Secondly, strategies to reduce FW should focus on optimizing supply chains and educating consumers about the efficient management of food resources.

Given these challenges, we believe that the UN’s strategy for combating FW must be strengthened and concretized through more efficient measures customized to the realities of each country. Customized action plans for each state should consider the economic, social, and cultural particularities that influence food resource management.

A critical aspect of this process is facilitating and encouraging food donations, a mechanism with significant potential for reducing waste. Uniform international legislation is necessary to regulate the legal framework for food donations and eliminate bureaucratic barriers that hinder the efficient redistribution of surplus food. Such an approach would contribute to reducing waste and facilitating access to essential food resources for vulnerable groups, transforming the issue of FW into an opportunity for solidarity and sustainability.

### 5.3. Limitations and Further Research

This study focused exclusively on EU member states, which limits the generalizability of the results to other regions of the world. Future research could expand this framework by including more countries outside the EU to explore whether the identified models hold in different geographical and economic contexts. Expanding the sample size could allow for a more detailed analysis of regional and cultural differences.

Although the study analyzed five key variables, other factors that could significantly impact FW were not considered. For instance, cultural factors, such as dietary habits and social norms or factors related to waste management infrastructure, could provide a deeper understanding of the phenomenon. Future research could include educational levels, technological access, or psychological factors influencing consumption decisions.

This study used cross-sectional data, which limits the ability to analyze the evolution of FW and its influencing factors over time. Future research could adopt a longitudinal approach. Another research direction is the impact of public policies and private interventions on FW. It could be helpful to analyze how food price regulation policies or FW awareness campaigns influence consumer behavior and levels of waste. Our study highlighted the importance of approaches customized to local contexts in reducing FW. Future research could investigate how cultural, economic, and social factors unique to each region impact FW.

## 6. Conclusions

This study provided a comprehensive perspective on the factors influencing FW and the sustainability of food systems in the European Union, using advanced statistical analysis methods such as clustering, factorial analysis, a general linear model, and artificial neural networks (MLP). Through a multidimensional approach, the research highlighted the complexity of the relationships between economic, environmental, and social variables, offering a deeper understanding of the mechanisms underlying FW.

The results demonstrated that food prices and progress in achieving SDGs contribute to reducing FW, while retail food sales can contribute to its increase. Cluster analysis identified four distinct groups of countries, each with unique socioeconomic and sustainability characteristics, underscoring the diversity within the EU and the need for policies adapted to the specificities of each region.

This study contributed to the specialized literature by integrating complex analysis methods and emphasizing the importance of multidimensional approaches in understanding FW. It also opened new research directions, such as expanding the sample size, including additional variables, and exploring more advanced analysis methods.

This research highlights the importance of ongoing research efforts and fostering collaboration among researchers, policymakers, and the private sector to address the complex challenges of FW and promote more sustainable and resilient food systems. By combining scientific knowledge with innovative practices, we can contribute to a more sustainable and equitable future for all.

## Figures and Tables

**Table 1:** Research variables.

Variable	Dataset	Measures	References
HICP	HICP food—annual data	Annual average index	[64]
FW	Food waste	Kilograms per capita	[1]
RSF	Retail sale of food	Index, 2021 = 100	[65]
SDGi	Sustainable Development Goals Index	Score	[66]
GDPpc	GDP per capita in PPS	Volume indices of real expenditure per capita (in PPS_EU27_2020 = 100)	[67]

Source: author’s design based on [1,64,65,66,67].

**Table 2:** Descriptive statistics.

Variable	Min	Max	Mean	Std. Deviation	Skewness	Kurtosis
HICP	92.5	163.5	116.412	12.4735	1.247	2.339
FW	65.0	294.0	137.173	47.2149	1.232	2.281
RSF	91.3	124.2	102.062	6.7653	0.992	0.841
SDGi	72.5	86.8	80.125	3.2049	−0.027	0.184
GDPpc	57	260	104.00	44.138	2.214	5.047

Source: developed by authors using SPSS v27.

**Table 3:** Correlation matrix, KMO, and Bartlett’s test.

		HICP	FW	RSF	SDGi
Correlation	HICP	1.000	−0.251	0.750	−0.101
	FW	−0.251	1.000	−0.052	−0.307
	RSF	0.750	−0.052	1.000	0.016
	SDGi	−0.101	−0.307	0.016	1.000
Sig. (1-tailed)	HICP		00.012	0.000	0.184
	FW	0.012		0.324	0.003
	RSF	0.000	0.324		0.445
	SDGi	0.184	0.003	0.445	
Kaiser-Meyer-Olkin Measure of Sampling Adequacy				0.392	
Bartlett’s Test of Sphericity		Approx. Chi-Square		87.880	
		df		6	
		Sig.		0.000	

Source: author’s design using SPSS v.27.0 (IBM Corporation, Armonk, NY, USA).

**Table 4:** Communalities and component matrix.

	Initial	Extraction	Component	
			1	2
HICP	1.000	0.897	0.937	0.139
FW	1.000	0.673	−0.362	0.736
RSF	1.000	0.833	0.893	0.187
SDGi	1.000	0.691	0.037	−0.830
Extraction Method: Principal Component Analysis.				

Source: author’s design using SPSS v.27.0 (IBM Corporation, Armonk, NY, USA).

**Table 5:** Total variance explained.

Factor	Initial Eigenvalues			Extraction Sums of Squared Loadings		
	Total	% of Variance	Cumulative %	Total	% of Variance	Cumulative %
1	1.808	45.192	45.192	1.808	45.192	45.192
2	1.286	32.139	77.330	1.286	32.139	77.330
3	0.711	17.778	95.109			
4	0.196	4.891	100.000			

Source: author’s design using SPSS v.27.0 (IBM Corporation, Armonk, NY, USA).

**Table 6:** Tests of between-subjects effects.

Source	Type III Sum of Squares	df	Mean Square	F	Sig.	Partial Eta Squared	Observed Power
Corrected Model	42,714.808	3	14,238.269	8.084	0.000	0.240	0.989
Intercept	29,152.726	1	29,152.726	16.551	0.000	0.177	0.980
HICP	25,536.469	1	25,536.469	14.498	0.000	0.158	0.964
RSF	11,606.540	1	11,606.540	6.590	0.012	0.079	0.717
SDGi	23,922.661	1	23,922.661	13.582	0.000	0.150	0.953
Error	135,624.772	77	1761.361				
Total	1,702,467.000	81					
Corrected Total	178,339.580	80					

Source: author’s design using SPSS v.27.0.

**Table 7:** GLM parameters.

Parameter	B	Std. Error	t	Sig.	95% Confidence Interval		Partial Eta Squared	Observed Power
					Lower Bound	Upper Bound		
Intercept	554.472	136.290	4.068	0.000	283.084	825.860	0.177	0.980
HICP	−2.196	0.577	−3.808	0.000	−3.345	−1.048	0.158	0.964
RSF	2.716	1.058	2.567	0.012	0.609	4.824	0.079	0.717
SDGi	−5.477	1.486	−3.685	0.000	−8.436	−2.518	0.150	0.953

Source: author’s design using SPSS v.27.0.

**Table 8:** Parameter estimates.

Parameter Estimates					
		Hidden Layer 1	Output Layer	Importance	Normalized Importance
		H (1:1)	FW		
Input Layer	(Bias)	1.881			
	HICP	1.054		0.396	76.7%
	RSF	−0.266		0.088	17.0%
	SDGi	1.306		0.516	100.0%
Hidden Layer 1	(Bias)		1.175		
	H (1:1)		−2.415		

Source: authors’ design using SPSS v.27 (IBM Corporation, Armonk, NY, USA).

## Data Availability

Data are available in a publicly accessible repository. The data presented in this study are openly available in: https://ec.europa.eu/eurostat/databrowser/view/prc_hicp_aind/default/table?lang=en&category=prc.prc_hicp (accessed on 16 March 2025); https://ec.europa.eu/eurostat/databrowser/view/env_wasfw/default/table?lang=en (accessed on 16 March 2025); https://ec.europa.eu/eurostat/databrowser/view/sts_trtu_a__custom_15765134/default/table?lang=en (accessed on 16 March 2025); https://dashboards.sdgindex.org/static/downloads/files/SDR2024-data.xlsx (accessed on 16 March 2025); https://ec.europa.eu/eurostat/databrowser/view/tec00114/default/table?lang=en (accessed on 16 March 2025).

## Abbreviations

The following abbreviations are used in this manuscript:

HICP	HICP food—annual data
FW	Food waste
RSF	Retail sale of food
SDGi	Sustainable Development Goals Index
GDPpc	GDP per capita in PPS

## Appendix A

**Table A1:** Clusters.

Cluster	Country	HICP	FW	RSF	SDGi	GDPpc
1	Austria	120.68	131	104.7	82.28	123
1	Belgium	117.74	151	102.0	79.46	119
1	Estonia	141.13	134	112.4	81.68	84
1	France	117.74	139	106.8	82.05	98
1	Germany	129.2	129	105.4	83.36	118
1	Ireland	99.0	144	101.0	80.15	238
1	Italy	116.8	139	104.5	78.79	98
1	Latvia	143.50	124	116.0	80.68	69
1	Lithuania	147.77	141	114.0	76.81	88
1	Luxembourg	120.58	122	104.8	77.65	252
1	Netherlands	122.73	129	104.1	79.42	134
1	Poland	140.0	123	124.2	81.80	78
1	Sweden	124.99	117	112.5	85.98	115
Cluster 1 means		126.30	132.54	108.65	80.78	124.15
2	Bulgaria	147.58	95	113.4	74.62	62
2	Croatia	124.21	72	113.4	81.50	72
2	Czechia	135.7	101	108.4	81.87	89
2	Finland	113.10	109	103.8	86.76	107
2	Hungary	163.54	84	119.0	79.39	77
2	Slovakia	137.50	106	113.9	79.12	71
2	Slovenia	125.03	71	111.5	81.01	89
2	Spain	122.72	65	108.6	80.43	88
Cluster 2 means		133.67	87.88	111.50	80.59	81.88
3	Cyprus	113.83	294	108.4	72.49	98
3	Denmark	119.7	254	103.3	85.68	135
Cluster 3 mean		116.77	274.00	105.85	79.09	116.50
4	Greece	115.93	195	107.9	78.37	67
4	Malta	129.18	162	112.2	75.53	105
4	Portugal	120.09	184	110.8	80.02	77
4	Romania	138.81	181	116.4	77.46	74
Cluster 4 mean		126.00	180.50	111.83	77.84	80.75
EU means		127.73	136.89	109.76	80.16	104.63

Source: authors’ design using SPSS v.27 (IBM Corporation, Armonk, NY, USA).
